# Spontaneous femoral neck fracture resulting from osteonecrosis involving lateral femoral head-neck junction: a retrospective study

**DOI:** 10.1186/s12891-023-07058-6

**Published:** 2023-11-27

**Authors:** Xin Guo, Yihui Zeng, Haijun Xu, Xinyuan Zhan

**Affiliations:** 1 Department of Orthopedics, Wuhan Fourth Hospital, Wuhan, China; 2 Department of Radiology, Wuhan Fourth Hospital, Wuhan, China; 3 Department of Anesthesiology and Pain Medicine, Tongji Hospital, Tongji Medical College, Huazhong University of Science and Technology, Wuhan, 430000 China

**Keywords:** Femoral neck fracture, Spontaneous fracture, Osteonecrosis, Osteoporosis

## Abstract

**Background:**

Spontaneous femoral neck fracture is a rare condition that remains controversial due to limited reported cases. This retrospective study aims to provide further insights into the etiology and characteristics of the disease.

**Method:**

We conducted a retrospective review of data from 963 patients with femoral neck fractures. The data encompassed demographic information, medical histories, radiographic records, bone mineral density (BMD) measurements, and pathological examinations. Patients were categorized into two groups: spontaneous femoral neck fracture (SFF) group (30 cases) and control group (933 cases), based on their medical histories. Logistic regression analysis was employed to identify risk factors for SFF. Statistical analysis was performed to compare and elucidate the characteristics of SFF within each group.

**Results:**

Logistic regression analysis revealed osteonecrosis of the femoral head, steroid use, and osteoporosis as three significant risk factors for SFF. Furthermore, a higher proportion of Garden type I and II fractures, as well as Pauwels type I fractures, were observed in the SFF group compared to the control group. Within the SFF group, a higher proportion of patients with osteonecrosis exhibited Garden type III and IV fractures compared to those with osteoporosis. Additionally, both magnetic resonance imaging (MRI) and pathological examinations demonstrated that osteonecrosis in the SFF group predominantly occurred at the lateral femoral head-neck junction.

**Conclusions:**

Osteonecrosis of the femoral head, particularly involving the lateral head-neck junction, was confirmed as a major risk factor for SFF. Furthermore, SFF exhibits internal heterogeneity based on its different causes.

**Supplementary Information:**

The online version contains supplementary material available at 10.1186/s12891-023-07058-6.

## Introduction

Spontaneous femoral neck fractures (SFFs) are fractures of the femoral neck that occur without any trauma or sudden accidents. They are rare in clinical practice, with only a few reported cases. Previous studies have primarily attributed the cause of SFFs to osteoporosis [1], osteopathy [2], or excessive stress [3, 4], which compromise the structural integrity of the trabecular bone in the femoral neck. Typically, these fractures present as incomplete fractures involving the femoral neck. However, we have encountered a few patients who did not have a clear history of trauma or intense hip exercises, and who were not diagnosed with osteoporosis or malignancy. Moreover, most of these fractures were complete fractures involving the subcapital area and classified as Garden type III, unlike typical SFFs, which are incomplete fractures classified as Garden type I. These cases differed not only in etiology but also in imaging characteristics from the usual SFFs and femoral neck stress fractures. Similar cases have been reported by several studies [5, 6], and insidious osteonecrosis at the lateral part of the femoral head-neck junction has been proposed as the main cause [6]. Although it is reasonable to consider insidious osteonecrosis of the femoral head (ONFH) as one of the etiologies of SFFs, with the mechanism being clear — osteonecrosis weakening the femoral neck, leading to stress-concentrated SFFs — there is a scarcity of similar studies and insufficient sample sizes to support the hypothesis or provide a comprehensive overview of SFFs caused by osteonecrosis. Therefore, we conducted a retrospective study with a larger sample size to determine whether osteonecrosis in the femoral head-neck region is an independent risk factor for SFFs and to identify the distinct features of SFFs caused by osteonecrosis compared to those caused by other etiologies.

## Patients and methods

A total of 963 patients (963 hips) who presented with femoral neck fractures at our hospital between January 2020 and January 2023 were included in the study. The data of each patient were reviewed and analyzed. The inclusion criteria were as follows:

1. Diagnosed with femoral neck fractures not combined with other fractures.
2. Over 18 years old and mentally competent.
3. Within 14 days of the injury.

Patients with the following conditions were excluded:

1. Patients without a definitive medical history.
2. Patients with autoimmune diseases causing osteoporosis.
3. Patients diagnosed with tumors prone to bone metastasis.

All 963 patients were informed that their case data might be used for publication, and ethical approval with informed consent was obtained from each patient. The study was approved by the ethics committee of Wuhan Fourth Hospital (No. KY2023-011-01).

Demographic information and medical history were collected from the patients’ admission records. Follow-up records were also consulted to enhance the accuracy of the information.

All patients underwent pelvic and femoral neck radiographs. The Garden and Pauwels classifications were recorded based on the radiographs. Magnetic resonance imaging (MRI) of the hip was available for 171 patients, including 30 with SFFs. Bone mineral density (BMD) measurements using dual-energy X-ray absorptiometry (DEXA) were available for 834 patients. A diagnosis of osteoporosis was made if the T-score of the lumbar spine was less than − 2.5. The T-score of the proximal femur was not considered, despite its higher accuracy in diagnosing osteoporosis, to minimize bias caused by femoral neck lesions. For patients who underwent hip arthroplasty, femoral heads (487 cases) were fixed with a 10% buffered formalin solution and embedded in paraffin. Coronal sections were examined using hematoxylin-eosin (HE) and Movat pentachrome staining.

The patients were divided into two groups: the SFF group and the control group, based on whether there was a definite history of trauma prior to the fracture. Within the SFF group, further division was made into the SFF group with osteonecrosis and the SFF group without osteoporosis to mitigate bias related to osteoporosis.

Age, height, and weight values are presented as mean ± SD. Student’s t-test was employed for continuous variables, while the Chi-squared test was used to compare binary variables. Logistic regression was conducted to identify the risk factors associated with SFFs. The covariates used in the logistic regression analysis included age (< 30 = 1; 30 ~ < 50 = 2; 50 ~ < 70 = 3; >70 = 4), gender (male = 1, female = 2), BMI (< 24 = 1; 24 ~ < 28 = 2; ≥28 = 3), osteoporosis (T-score ≥ -1 = 1; -1 > T-score ≥ -2.5 = 2; T-score < -2.5 = 3), osteonecrosis of the femoral head (negative = 0; positive = 1), and steroid use (negative = 0; positive = 1). All statistical analyses were performed using SPSS 25.0 (SPSS Inc., Chicago, IL, USA).

## Results

Among the patients, 312 were males and 651 were females. The average age at the time of in-patient admission was 67.8 ± 13.2 years (range: 16–97 years). Out of the 963 patients, 549 (57.0%) underwent hip arthroplasty (total hip arthroplasty [THA] and hemiarthroplasty), 269 (27.9%) received closed reduction and internal fixation, and the remaining 145 (15.1%) were treated nonoperatively.

Within the 963 patients, 30 experienced spontaneous femoral neck fractures without a history of trauma. The average age of these patients was 62.3 ± 7.4 years (range: 40–73 years). Among the 30 patients, 8 were diagnosed with osteonecrosis and 12 with osteoporosis. Detailed demographic information for each group is presented in Table 1. Binary logistic regression was utilized to analyze the risk factors associated with SFFs. The results revealed that osteoporosis, steroid use, and ONFH were three independent risk factors for SFFs. Age, gender, and BMI were not identified as risk factors for SFFs.

**Table 1:** Baseline characteristics of all patients, the group with SSF and the control group

	Patients in total	SFF group			The control group		
		**total**	**With osteonecrosis**	**With osteoporosis**	**total**	**With osteonecrosis**	**With osteoporosis**
Cases	963	30	8	12	933	6	132
Gender (male/female)	312/651	12/18	4/4	0/12	299/634	3/3	25/107
Age (year)	67.8 ± 13.2	63.0 ± 13.4	66.9 ± 7.5	70.3 ± 10.7	68.0 ± 13.1	52 ± 11.8	70.4 ± 11.9
BMI	25.9 ± 2.7	24.7 ± 2.7	24.3 ± 3.2	24.4 ± 2.5	25.6 ± 2.7	23.4 ± 2.0	26.2 ± 3.2
Garden type							
I	38(3.9%)	4(13.3%)	0	1(8.3%)	36(3.9%)	0	0
II	116(12.1%)	14(46.7%)	2(25.0%)	8(66.7%)	106(11.3%)	0	7(5.3%)
III	427(44.3%)	8(26.7%)	3(37.5%)	2(16.7%)	419(44.9%)	4(66.7%)	52(39.4%)
IV	382(39.7%)	4(13.3%)	3(37.5%)	1(8.3%)	372(39.9%)	2(33.3%)	73(55.3%)
Pauwels type							
I	48(5.0%)	20(66.7%)	4(50.0%)	10(83.3%)	32(3.4%)	0	19(14.4%)
II	683(70.9%)	10(33.3%)	4(50.0%)	2(16.7%)	669(71.7%)	6(100%)	77(58.3%)
III	232(24.1%)	0	0	0	232(24.9%)	0	26(17.4)

When focusing specifically on SFFs, no statistically significant differences were observed in age, BMI, or gender between the SFF group and the control group. However, significant differences in the distribution of Garden types (Fisher’s exact test: P < 0.01) and Pauwels types (Fisher’s exact test: P < 0.01) were observed between the two groups. Furthermore, when examining the SFF group based on different risk factors, radiographs revealed variations in SFF presentations. The proportion of non-displaced fractures (Garden I and II) was significantly lower in SFFs with osteonecrosis compared to SFFs with osteoporosis (Fisher’s exact test: P < 0.01). This suggests that SFFs result from diverse pathogenic mechanisms, resulting in distinct clinical manifestations.

**Fig. 1:**
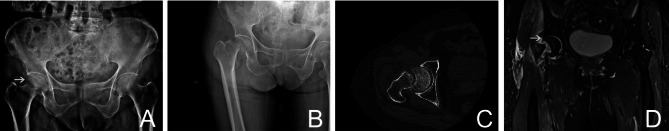
The imaging performance of a 77-year-old woman who presented with SFF with osteonecrosis involving lateral femoral head-neck junction. **A**, the radiograph 10 days before the SFF occurred. A low density zone appeared at the right lateral femoral head-neck junction, but there were no occult fractures. **B**, the radiograph after the SFF occurred. **C**, the CT (computerized tomography) presentation. **D**, the T2 weighted image of MRI showed high signal around the femoral head-neck junction

**Fig. 2:**
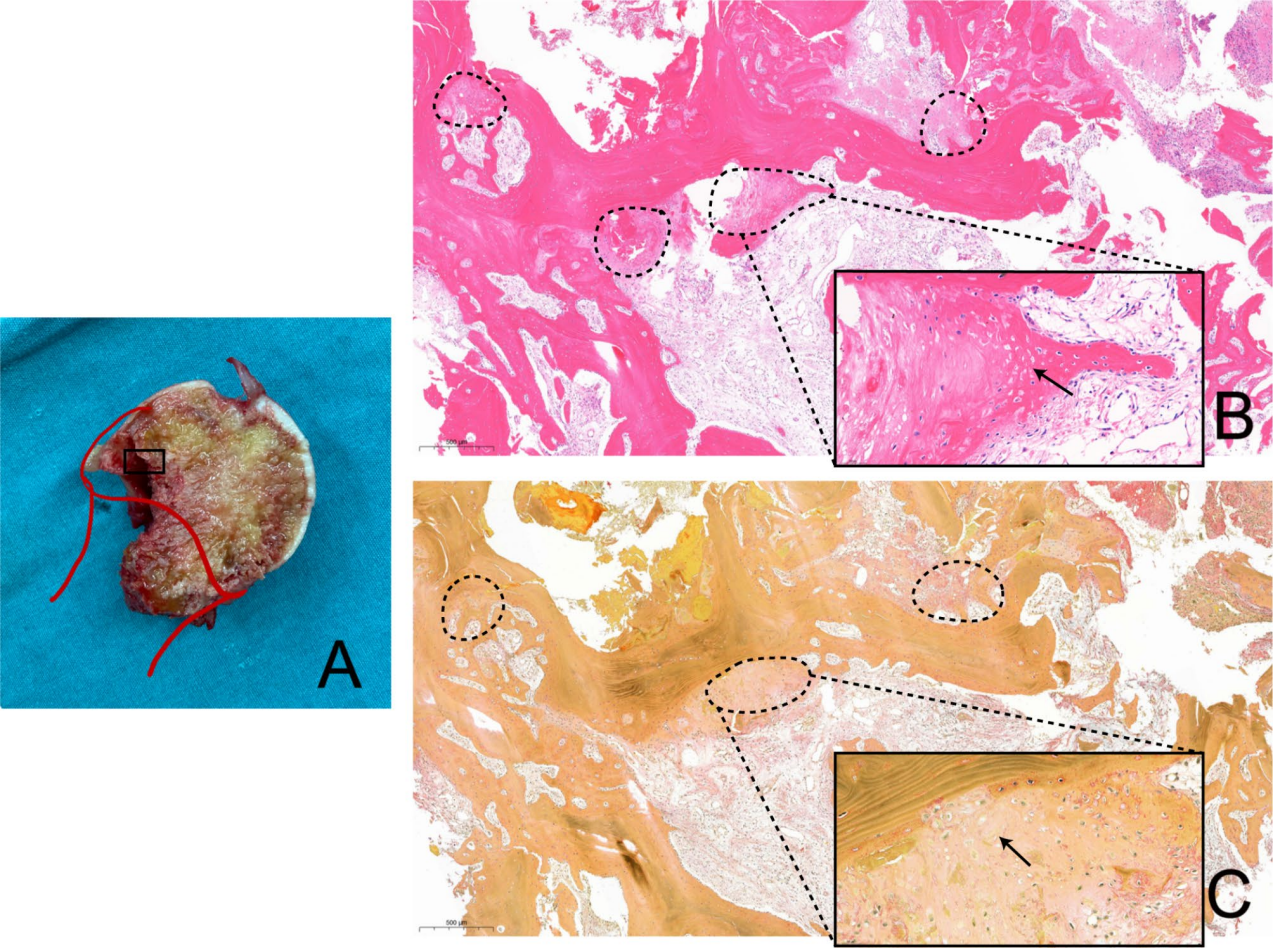
At the femoral head-neck junction (**A**), the HE (**B**) and Movat pentachrome (**C**) stain showed osteonecrosis (indicated by dotted line) dispersed among trabeculae partially. The necrotic tissue was surrounded by inflammatory cells and fibroblastic cells. The dead bone cells were indicated with arrows

Using hip MRI, the positions of osteonecrosis were determined. In all eight patients in the SFF group with osteonecrosis, osteonecrosis involving the lateral femoral head-neck junction was observed (Fig. 1). In contrast, only one out of the six patients in the control group with osteonecrosis had osteonecrosis involving the lateral femoral head-neck junction. Pathological examination confirmed the presence of osteonecrosis in histological samples. Tissues from the SFF group with osteonecrosis exhibited a typical pattern of dead osteocytes in the lateral trabeculae of the femoral head-neck junction, along with a zonal pattern displaying areas of bone infarction, reparative granulation tissue, and viable tissue, consistent with a pathological diagnosis of ONFH (Fig. 2).

## Discussion

In this study, we aimed to collect and investigate a substantial number of cases of femoral neck fractures in order to obtain a sufficient sample size for SFFs. By including a larger number of SFF cases than previous articles, we were able to gain further insights into the causes, pathogenesis, and clinical manifestations of SFF. Logistic regression analysis revealed that ONFH, osteoporosis, and steroid use were confirmed as the three major and independent risk factors for SFF. Among these factors, ONFH carried the highest weight, indicating that although ONFH is rare, patients with this condition are more prone to SFF compared to those with osteoporosis or steroid use. Therefore, it is crucial to pay closer attention to patients with ONFH, particularly when it involves the head-neck junction. Our results indicated that SFFs primarily occurred during the Stage II progression of ONFH according to the ARCO Classification System, which is before the collapse and depression of the femoral head (Stage III). Generally, hip preservation treatments are suitable for Stage II ONFH, core decompression, femoral osteotomy and vascularized bone-grafting have proven effective for pre-collapse lesion [7]; however, once SFF occurs, there are limited treatment options available, with THA being the most common choice. Osteoporosis was another risk factor for SFF according to the results. It is reasonable that, after minerals drain from the bone, SFF occurred even though slight stress is exerted on the femoral neck. As for steroid use, the pathogenesis is obscure. A possible mechanism is that steroid use leads to ONFH and osteoporosis, both of which give rise to SFF subsequently, but the results of logistic regression analysis suggest that there must be other factors that function on SFF independently hidden behind steroid use, that needs to be studied further.

The radiological manifestations of SFFs exhibit both similarities and differences depending on the underlying causes. Regarding the Garden classification, SFFs associated with osteonecrosis have a higher proportion of displaced fractures (Garden types three and four) compared to those with osteoporosis (75% versus 25%). On the other hand, in terms of the Pauwels classification, SFFs, as well as cases of osteonecrosis and osteoporosis, were not classified as Pauwels type three. This suggests that minor trauma acting on a compromised femoral neck is the primary cause of SFFs, and the causative factors only determine the severity of bone destruction. The similarity in Pauwels type implies low-energy trauma, while the difference in Garden type indicates that osteonecrosis weakens the femoral neck more than osteoporosis.

Biopsy and MRI revealed a higher incidence of ONFH in the lateral region of the femoral head-neck junction in SFF patients. Although osteonecrosis can occur in any part of the femoral head [7], it typically originates from weight-bearing areas other than the lateral region of the femoral head-neck junction. This suggests that ONFH in the lateral region, as observed in MRI, may serve as an early warning sign for SFF. Furthermore, the pathogenesis and radiological classifications of SFF can be explained by lesions in the lateral region. Osteonecrotic lesions originating from or involving the lateral femoral head-neck junction leads to trabecular destruction and compromised structural integrity. Consequently, even minor injuries or normal muscle contractions can result in fractures. Due to the normalcy of the medial part of the femoral neck, the lateral part is more prone to displacement, thereby explaining the higher occurrence of Garden type III fractures. Additionally, the fracture lines tend to follow the arc of the femoral head, which parallels the distribution of osteonecrosis. This likely explains why none of the SFF cases with osteonecrosis belonged to Pauwels type III.

Several limitations should be acknowledged in this research. Firstly, the collection of medical history from each patient lacked standardization. The verification of spontaneous femoral neck fractures relied solely on subjective patient statements, which may introduce subjectivity and instability in statistical analysis, despite our efforts to collect detailed medical histories. Secondly, this study adopted a retrospective design. While our findings clearly demonstrated the close association of ONFH, steroid use, and osteoporosis with SFFs, it remains challenging to establish these factors as direct causes of SFF due to the potential presence of confounding biases that are difficult to entirely eliminate. Finally, although fatigue stress fracture has been reported as a cause of SFF [4, 8, 9], we did not include this factor in our analysis due to incomplete collection of relevant medical history. The inclusion of fatigue stress fractures would have strengthened the study and increased its overall credibility.

Is it possible to prevent SFF? Among the three risk factors of SFF, osteoporosis and steroid use are easier to be prevented. While, ONFH is an idiopathic disease hard to be found and controlled at the early stage. So, orthopedic surgeons should pay more attention to the slight symptoms as well as radiological manifestations at the hip for early detection of SFF in clinical field.

## Conclusions

Our findings indicate that SFF is not a disease resulting from a single etiology. In addition to osteoporosis, occult osteonecrosis at the lateral region of the femoral head-neck junction serves as another independent cause of SFF. Depending on the underlying causes, SFFs exhibit distinct clinical presentations and imaging features.

### Electronic supplementary material

Below is the link to the electronic supplementary material.

**Supplementary Material 1:** The results of binary logistics regression: the variables in the equation.

## Data Availability

The datasets used and analyzed during the current study are available from the corresponding author on reasonable request.

## Abbreviations

BMD: Bone mineral density
SFF: Spontaneous femoral neck fracture
MRI: Magnetic resonance imaging
ONFH: Osteonecrosis of the femoral head
DEXA: Dual-energy X-ray absorptiometry
HE staining: Hematoxylin-eosin staining
THA: Total hip arthroplasty
