# Supplementary material for: Spontaneous femoral neck fracture resulting from osteonecrosis involving lateral femoral head-neck junction: a retrospective study
Source: BMC Musculoskelet Disord. 2023 Nov 27;24:913. doi: 10.1186/s12891-023-07058-6 (PMC10680177; doi:10.1186/s12891-023-07058-6)
Supplement: Supplementary file 1 — Supplementary Material 1: The results of binary logistics regression: the variables in the equation. [file 12891_2023_7058_MOESM1_ESM.docx]

The results of binary logistics regression: the variables in the equation.

|  | **B** | **S. E.** | **Wald** | **df** | **Sig.** | **Exp(B)** |
| --- | --- | --- | --- | --- | --- | --- |
|  |  |  |  |  |  |  |
| **ONFH** | 4.740 | .721 | 43.164 | 1 | .000 | 114.418 |
| **Steroids use** | 2.788 | .498 | 31.280 | 1 | .000 | 16.243 |
| **Osteoporosis** | 1.403 | .456 | 9.468 | 1 | .002 | 4.067 |
| **constant** | -4.424 | .336 | 173.802 | 1 | .000 | .012 |
